# Supplementary figures and images for: Pericystic brain transcriptomics reveals molecular signatures of immune activation and neurovascular remodelling in viable and post-treatment porcine neurocysticercosis
Source: bioRxiv. 2026 Jul 1:2026.06.26.734379. Preprint. [Version 1] doi: 10.64898/2026.06.26.734379 (PMC13345202; doi:10.64898/2026.06.26.734379)

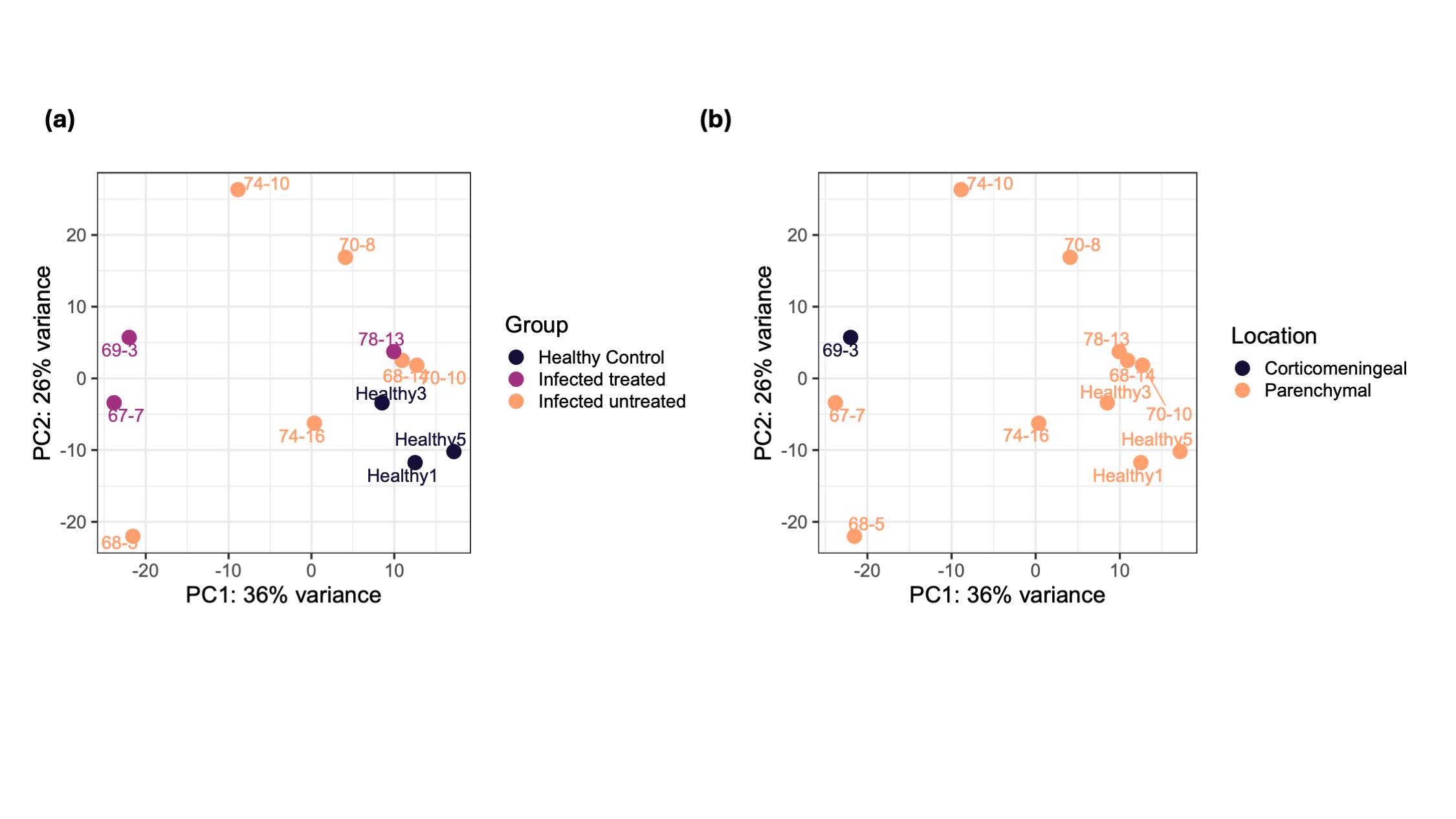

Supplement: Supplement 1 — Figure S1. Principal component analysis of samples. PCA plot based on variance-stabilized gene expression data, showing the distribution of samples based on (a) experimental group and (b) location of the sample. Supplementary Table 1. DESeq2 differential expression results for the infection effect. Complete gene-level output from the comparison between infected untreated and uninfected control samples, including log2 fold changes, test statistics, nominal p-values and adjusted p-values. Supplementary Table 2. KEGG pathways significantly enriched for the infection effect. Gene set enrichment analysis results for the infection-effect comparison, including pathways with absolute normalised enrichment score (|NES|) > 1 and false discovery rate (FDR) < 0.05. Supplementary Table 3. DESeq2 differential expression results for the treatment effect. Complete gene-level output from the comparison between treated infected and untreated infected samples, including log2 fold changes, test statistics, nominal p-values and adjusted p-values. Supplementary Table 4. KEGG pathways significantly enriched for the treatment effect. Gene set enrichment analysis results for the treatment-effect comparison, including pathways with absolute normalised enrichment score (|NES|) > 1 and false discovery rate (FDR) < 0.05. [file media-1.zip › Supplementary information/Fig S1.tiff]
